# De Novo Drug Design Using Transformer-Based Machine Translation and Reinforcement Learning of an Adaptive Monte Carlo Tree Search

**DOI:** 10.3390/ph17020161

**Published:** 2024-01-27

**Authors:** Dony Ang, Cyril Rakovski, Hagop S. Atamian

**Affiliations:** 1 Computational and Data Sciences Program, Chapman University, Orange, CA 92866, USA; doang@chapman.edu (D.A.); rakovski@chapman.edu (C.R.); 2 Schmid College of Science and Technology, Chapman University, Orange, CA 92866, USA; 3 Biological Sciences Program, Chapman University, Orange, CA 92866, USA

**Keywords:** artificial intelligence, drug design, novel molecules, encoder–decoder, transformer, quantitative estimate of drug-likeness (QED), virtual screening, validity, reinforcement learning, molecular docking

## Abstract

The discovery of novel therapeutic compounds through de novo drug design represents a critical challenge in the field of pharmaceutical research. Traditional drug discovery approaches are often resource intensive and time consuming, leading researchers to explore innovative methods that harness the power of deep learning and reinforcement learning techniques. Here, we introduce a novel drug design approach called drugAI that leverages the Encoder–Decoder Transformer architecture in tandem with Reinforcement Learning via a Monte Carlo Tree Search (RL-MCTS) to expedite the process of drug discovery while ensuring the production of valid small molecules with drug-like characteristics and strong binding affinities towards their targets. We successfully integrated the Encoder–Decoder Transformer architecture, which generates molecular structures (drugs) from scratch with the RL-MCTS, serving as a reinforcement learning framework. The RL-MCTS combines the exploitation and exploration capabilities of a Monte Carlo Tree Search with the machine translation of a transformer-based Encoder–Decoder model. This dynamic approach allows the model to iteratively refine its drug candidate generation process, ensuring that the generated molecules adhere to essential physicochemical and biological constraints and effectively bind to their targets. The results from drugAI showcase the effectiveness of the proposed approach across various benchmark datasets, demonstrating a significant improvement in both the validity and drug-likeness of the generated compounds, compared to two existing benchmark methods. Moreover, drugAI ensures that the generated molecules exhibit strong binding affinities to their respective targets. In summary, this research highlights the real-world applications of drugAI in drug discovery pipelines, potentially accelerating the identification of promising drug candidates for a wide range of diseases.

## 1. Introduction

The task of finding molecules that bind to specific biological targets is challenging due to the vast molecular space [1]. Despite recent advancements in high-throughput screening methods, screening millions of molecules for their binding to proteins of interest remains a costly and time-consuming process [2]. The possible promiscuity of the identified molecules poses yet another challenge that is receiving increasing attention in drug discovery [3]. Molecules can bind to multiple off targets, leading to undesirable and sometimes even life-threatening side effects [4]. Yet, molecules that target multiple biological targets simultaneously, could be needed for treating complex illnesses such as cancer and cardiovascular disease. Such promiscuous drugs, such as aspirin, are very rare but very effective [5]. This biological complexity could be addressed by the integration of cutting-edge data science that incorporates AI-driven approaches and by encouraging collaboration between academia, pharmaceutical companies, and the biotechnology industry.

Computational methods have played a pivotal role in accelerating drug discovery and reducing associated costs [6]. By combining ligand- and structure-based virtual screening methods, researchers can efficiently screen large chemical databases to identify potential drug candidates in a short time [7]. Furthermore, scientists can employ molecular dynamics simulations to predict how molecules will interact with specific biological targets [8]. These methods, although effective, have limitations as they rely on existing molecules, whether synthetic or natural. As part of the recent wave of advancements in artificial intelligence, generative AI models have emerged as a powerful tool in the field of drug discovery, particularly for de novo drug design, which involves creating novel molecular structures [9].

Generative AI models used in drug discovery can be broadly categorized into two main groups based on their use of target information. The models in the first category are trained on a large dataset of known molecules, allowing them to efficiently explore the chemical space and generate molecules with properties similar to known compounds. However, this focus on similarity to known molecules may limit their ability to produce entirely novel chemical structures and optimize interactions with specific targets. In the second category, models rely on the 3D structure or binding site characteristics of the target, enabling the generation of molecules tailored for interaction with specific targets. Nevertheless, a major drawback of such models is the limited availability of high-quality structural data for many drug targets [10]. To leverage the advantages of both categories, a combination of these models is often used.

Self-supervised pretraining, which involves training models on large amounts of unlabeled data, has become a dominant paradigm in Natural Language Processing (NLP) and has been successfully used in the two influential NLP technologies GPT and BERT [11,12]. Researchers have adapted the principles of NLP pretraining to develop “chemical language models” given the analogies between human language and the “language” of chemical structures represented by SMILES (Simplified Molecular Input Line Entry System) strings. In natural languages, grammatical rules and syntax govern the arrangement of words in sentences, which affects the meaning and interpretation of text [13]. The chemical language follows similar specific rules and syntax to those in natural language for representing chemical structures, where the order and arrangement of symbols in SMILES strings convey information about the chemical structure and properties of a molecule. By treating chemical structures like sentences, where each symbol or combination of symbols represents a chemical entity (atom, bond, or group), chemical language models can be pre-trained on large datasets of SMILES strings. These models learn to generate SMILES strings by predicting each symbol or subsequent symbols [14]. Such chemical language models have been shown to obtain promising results on downstream tasks.

This research drew inspiration from Machine Translation (MT) using the sequence-to-sequence model [15] and from the rapid success of applying transformation architecture [16] in various NLP use cases. Greedy and beam search methods have traditionally been common approaches for decoding auto-regressive machine translation models in NLP [17,18]. Recently, the Monte Carlo Tree Search (MCTS)-based method has been demonstrated to outperform the greedy and beam search methods in generic language applications [19]. The MCTS is a probabilistic and heuristic-driven search algorithm that enables multiple constraint optimization steps. Moreover, this MCTS-based method has been successfully applied in Large Language Models (LLM) [20].

In this work, we introduce an innovative de novo drug design engine named drugAI, which marks the first integration of a decoder transformer model with the MCTS in the fields of bioinformatics and cheminformatics. At the core of drugAI, we employ an encoder–decoder transformer model coupled with a MCTS algorithm. This novel approach enabled us to implement multiple constraint optimization steps during the protein sequence to small molecule (SMILES string) generation. The drugAI engine, which was extensively trained on protein–ligand pairs, filtered from the comprehensive BindingDB [21], takes target protein sequences as input and generates small molecules (SMILES strings) as candidate inhibitors for these protein targets. Notably, drugAI surpasses traditional greedy and beam search methods by enabling multi-constrained optimization of the generated molecules. It evaluates their (1) validity; (2) pharmacological or biological properties for orally active drugs in humans [22]; (3) quantitative estimate of drug-likeness (QED) to gouge the compounds’ potential as drug candidates [23], and binding affinity to their targets. The molecules generated by drugAI were consistently valid with a validity rate of 100%. They showed 42% and 75% higher QED scores compared to those obtained through greedy and beam search methods, respectively. Furthermore, by integrating the binding affinity between the ligand and the target into the reinforcement learning process, the molecules generated by drugAI demonstrated strong binding affinities towards their respective targets. These affinities were comparable to those identified by traditional virtual screening approaches.

## 2. Results

### 2.1. Effectiveness of DrugAI

**Figure 1:**
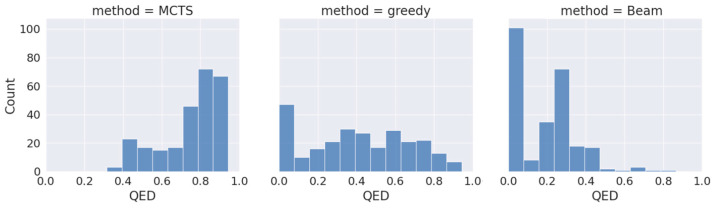
Distribution of QED drug-likeness of the generated molecules across the different decoding algorithms.

To assess the effectiveness of our proposed approach (drugAI) in generating small molecules with desired qualities for potential future drug candidates, we employed drugAI, alongside two other commonly used methods, greedy and beam, to train on the same dataset. We then conducted a comparative analysis of the results produced by these three methods by calculating various benchmarks within the GuacaMol codebase [24] using the Distributed-learning GuacaMol function. It is worth noting that the quantitative estimate of drug-likeness (QED) was not included in the standard GuacaMol benchmarks and was calculated separately using the RDKit package. DrugAI outperformed the greedy and beam methods by generating a significantly higher proportion of valid molecules (Table 1). In fact, all the molecules generated by drugAI were valid, whereas the greedy and beam methods produced 0.83 and 0.62 proportions of valid molecules, respectively. The uniqueness and novelty parameters of the generated molecules by drugAI were comparable to the greedy method but significantly higher than the beam method (Table 1). DrugAI demonstrated outstanding performance in terms of generating molecules with a high measure of drug-likeness, as assessed by the quantitative estimate of drug-likeness (QED). The molecules generated by drugAI achieved a significantly higher QED score (0.73) compared to greedy (0.41) and beam (0.18) (Table 1). The distribution of the QED scores also exhibited substantial differences. DrugAI did not produce QED scores below 0.3, while greedy search displayed scores spanning the entire spectrum. In contrast, the beam search predominantly lacked scores above 0.5 (Figure 1).

### 2.2. Physicochemical Properties of the Generated Molecules

**Figure 2:**
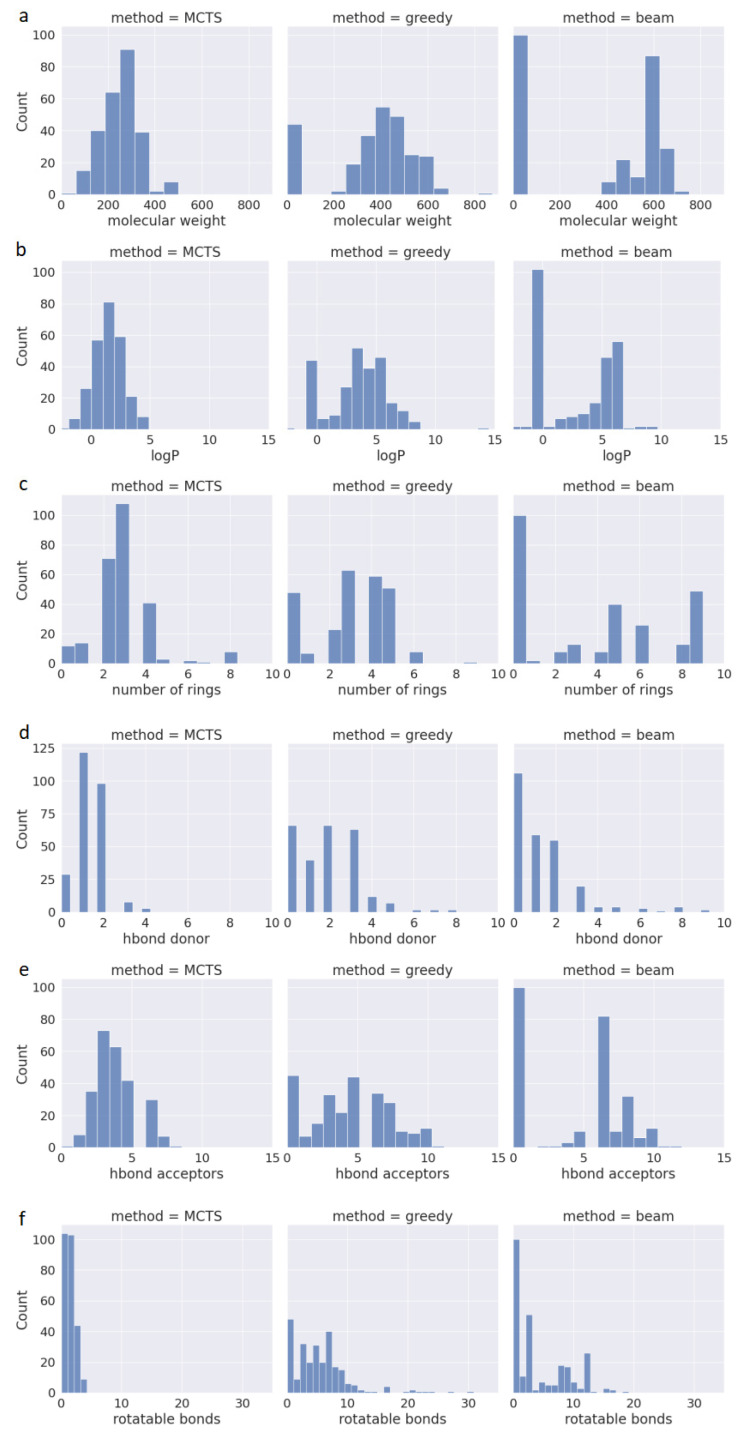
Distribution of properties for the generated molecules across the different decoding algorithms. (**a**) Molecular weight; (**b**) water–octanol partition coefficient (logP); (**c**) the number of rings; (**d**) the number of H donors; (**e**) the number of H acceptors; (**f**) the number of rotatable bonds.

One of the available methods for assessing the drug-likeness of a compound is Lipinski’s Rule of Five (also known as RO5). In addition to its effectiveness against a target protein, newly designed drugs should be suitable for oral administration. Therefore, RO5 predicts whether a chemical compound has favorable pharmacokinetic properties based on criteria such as Molecular Weight (MW < 500), Lipophilicity (LogP < 5), Hydrogen Bond Donors (HBD < 5), and Hydrogen Bond Acceptors (HBA < 10). Compounds meeting these criteria have a high likelihood of being orally bioavailable. However, it is worth mentioning that in drug discovery, no rule is absolute, as up to 10% of approved oral drugs violate RO5 [25]. Another key feature of drugAI, in addition to its ability to generate 100% valid molecules with high QED values, is its adherence to the RO5 criteria while generating molecules. This adherence serves as a valuable initial filter for identifying compounds with the potential for oral drug development. To evaluate the performance in terms of generating molecules that conform to the RO5 criteria, we calculated these metrics and assessed the physicochemical properties of the molecules generated by the three methods. Figure 2 presents the data regarding the compliance of the generated molecules from all three methods with the aforementioned rules. All the molecules generated by drugAI had logP values less than or equal to 5, whereas nearly one third of the molecules generated by greedy and beam searches exceeded this threshold, reaching as high as 15. Similarly, the molecular weight of the compounds generated by drugAI did not exceed the 500 threshold, while greedy and beam searches generated molecules with molecular weights of up to 800. Interestingly, unlike drugAI, both the greedy and beam methods produced a large number of very small molecules. Regarding HBD and HBA, drugAI strictly adhered to the RO5 criteria, producing molecules with values no higher than 5 for HBD and 10 for HBA, respectively. While HBA values rarely exceeded 10 in the greedy and beam methods, HBD values were more varied and often went beyond 5. The number of rotatable bonds was significantly smaller in the molecules generated by drugAI compared to the other two methods. There were no significant differences in terms of the number of rings among the three methods. In summary, these findings illustrate how drugAI can significantly enhance future de novo drug design efforts by generating drug-like molecules.

### 2.3. Demonstrating the Flexibility of DrugAI and Comparing It to Traditional Virtual Screening Approaches

Our findings conclusively demonstrated that drugAI outperforms two benchmark methods in terms of generating 100% valid molecules with high QED scores that are suitable for oral application. To further improve the model and showcase its adaptability and flexibility, we incorporated binding affinity (measured in kcal/mol) as an additional reward function in drugAI’s reinforcement learning. The binding affinity, which describes the binding strength between a drug molecule and its target, plays a significant role in early drug development. Thus, adding this fourth reward function would further enhance the quality of the molecules produced as potential drugs by ensuring that the generated molecules strongly bind to the target protein.

We previously discovered natural products that bind to the SARS-CoV-2 Main Protease (M^pro^) and inhibit its protease activity using a combined Ligand-based and Structure-based Virtual Screening (LBVS + SBVS) approach [26]. As a proof of concept, we used drugAI to generate small molecules targeting the same SARS-CoV-2 M^pro^ target protein. For consistency, both LBVS + SBVS and drugAI were configured to scan and utilize the same cavity coordinates and the same docking search area sizes [26]. As shown in Table 2, the average binding affinity for the top 10 generated molecules by drugAI is −9.4 (kcal/mol), which is comparable to the −9.37 (kcal/mol) obtained in [26]. The molecules generated by drugAI belonged to different chemical classes such as Benzenes, Isoindoles, Flavonoids, and Quinoles (Table S1). In conclusion, drugAI generated valid molecules with drug-like characteristics that are optimized to bind efficiently to their target in just two hours, with results comparable to widely used virtual screening methods, which take weeks to perform molecular docking on a large number of ligands.

## 3. Discussion

Generative machine learning models are designed to learn patterns and structures within existing data and create new, previously unseen data [27]. These models have gained popularity in drug discovery in recent years and are expected to revolutionize the future of pharmaceutical engineering [28]. The Encoder–Decoder Transformer architecture, which takes an input sequence and generates an output sequence, has been widely used in natural language processing (NLP) [16]. Recently, this Encoder–Decoder Transformer architecture has been adapted in the field of drug discovery, where it takes molecular structures or protein sequences as input and generates novel molecules or sequences. At the decoding step, which involves the process of generating new sequences or molecules by adding amino acids or atoms one at a time, a decision-making strategy is needed to make the best choice at each step based on selecting the token with the highest probability as the next token in the output sequence [29]. The two most popular decoding algorithms in sequence-to-sequence models are the greedy search and the beam search. Both are heuristic search algorithms that seek to find the most likely output sequence. While greedy search simply selects the token with the highest probability as the next token in the output sequence, beam search considers multiple candidates at each time step and retains a diverse set of candidates throughout the decoding process [17,18].

However, the challenge with these types of decoding algorithms in drug design is to ensure that the newly generated molecules adhere to certain constraints or properties that could make them successful drugs. Reinforcement learning (RL) has been successfully applied in fields such as speech recognition and formal languages to address this challenge by introducing value functions into the decoding mechanisms [30]. These value functions reward desired behaviors and penalize undesired ones [31]. In our approach, we employed the Monte Carlo Tree Search (MCTS) method to overcome some of the limitations of deep generative models that use greedy and beam searches. By utilizing value functions to assess validity, binding to target, and adherence to Lipinski’s Rule of Five (RO5), our generative model produced molecules that were both 100% valid and had significantly higher quantitative estimates of drug-likeness (QED) scores. Ensuring the generation of SMILES strings that are 100% chemically valid is crucial because it guarantees that all molecules to be investigated in the future can be chemically synthesized.

The QED, introduced almost a decade ago [23], is one of the most commonly used quantitative assessments of drug efficacy. Studies have shown that the average QED within the top cluster of human drug targets is 0.693. When focusing exclusively on the highest-ranked cluster of oral drug targets, the average QED increases to 0.766 [23]. This demonstrates that drugAI, with a mean QED of 0.73, is capable of generating molecules with properties suitable for potential oral drug candidates. In line with these findings, the analysis of the individual criteria within the RO5 showed that drugAI was able to accurately adhere to those criteria, which was not the case with other search methods. This, in itself, explains the superior QED scores of drugAI. In the future, additional benchmarks could be employed to further evaluate the molecules generated by drugAI.

Yet, another crucial advantage of drugAI is its ability to generate 100% novel molecules with very high QED scores. When computational programs aim to generate molecules with high drug-likeness, they tend to avoid exploring unconventional or entirely new chemical structures and often prioritize well-known molecular structures and properties that are associated with existing drugs. This often leads to difficulties in generating truly novel molecules because the algorithms tend to have a biased output toward known chemical patterns in the training set. The results from drugAI were remarkable in terms of balancing the generation of molecules with known drug-likeness properties (high QED value) and the exploration of novel chemical space as it generated 100% novel molecules, which shows that the model did not generate any molecules present in the training set. Thus, drugAI efficiently addressed the common challenge of “overfitting” in de novo drug design as it generated 100% novel SMILES strings, a result comparable to the greedy search. Deep learning models can sometimes memorize existing chemical structures rather than generate new ones, which often results in overfitting. In our case, drugAI was able to learn and generalize the chemical space to generate novel molecules.

Moreover, the model avoided generating the same molecule multiple times, as shown by the 84% uniqueness.

Many molecular docking techniques are available that can predict how molecules will bind to biomolecular targets and the affinity of this binding [32]. These virtual screening techniques have proven effective in finding new hits from extensive collections of chemical compounds, and in predicting their modes and affinities of binding [33]. Presently, molecular docking is a leading approach in the discovery of new compounds that act against target proteins. The flexibility of the MCTS-based reinforcement learning enabled us to incorporate this important matrix in the model. Accordingly, drugAI was able to generate molecules that showed strong binding affinity against the target comparable to those identified through virtual screening of large chemical databases. This showcases the capability of drugAI in the proficient and successful creation of potential drugs for various diseases in the future.

## 4. Materials and Methods

### 4.1. Data

The model was trained using experimentally determined protein–ligand binding affinities obtained from the BindingDB database. The complete database comprises over 2.4 million data records, offering a valuable source of information. To curate a focused dataset for training, we implemented selection criteria to extract the most relevant records. The following criteria were applied to filter the raw dataset:

1. The field “Target Source Organism According to Curator or DataSource” equals “Homo sapiens”;
2. The record has an IC50 value less than 100 nm; if the IC50 is missing, then Kd is less than 100 nm; if both are missing, then EC50 is less than 100 nm;
3. The record has SMILES representation.

This resulted in a dataset comprising 319,030 entries, consisting of 1298 unique amino acid sequences and 198,490 distinct ligand SMILES strings. To ensure uniformity and consistency, all SMILES strings used in this study were canonicalized using RDKit. The dataset was then randomly divided into two subsets: a training set, which comprised 70% of the data, and a test set, which made up the remaining 30%. The training subset consisted of 223,321 pairs of protein sequences and ligands, representing 1038 unique proteins. The test subset, on the other hand, contained 95,709 pairs of protein sequences and ligands, representing 260 unique proteins.

### 4.2. High-Level Architecture of DrugAI

**Figure 3:**
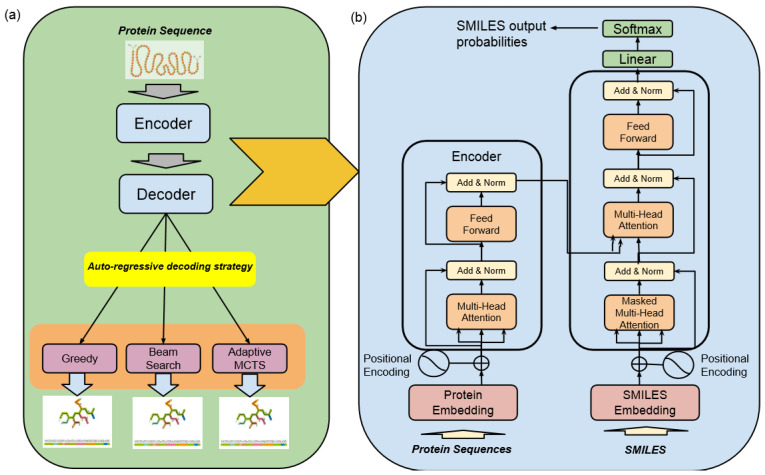
Machine translation—drugAI overview: (**a**) the workflow of the translation task of drugAI; (**b**) the encoder–decoder transformer architecture (modified from [16]).

The high-level architecture of the Encoder–Decoder Transformer, coupled with a Monte Carlo Tree Search (MCTS) for molecule generation, represents a state-of-the-art framework specifically designed for the discovery of highly effective lead molecule candidates targeted at specific receptor proteins in drug discovery, as illustrated in Figure 3.

This architecture combines two fundamental components to accelerate the process of identifying promising drug candidates:

- *Encoder–Decoder Transformer*
  At its core, this architecture employs a transformer model, which comprises an encoder and a decoder. The encoder takes input data in the form of protein sequences and transforms them into latent representations. Subsequently, the decoder utilizes these representations to systematically generate molecular sequences. The transformer model used in this study was trained with six layers of transformer blocks, each having a size of 512, a learning rate of 0.0001, and eight attention heads. The training process employed the Adam Optimizer with a batch size of five and a total of 25 epochs.
- *Monte Carlo Tree Search (MCTS)*
  The MCTS is a heuristic search algorithm used in conjunction with the transformer model. It facilitates the exploration of the vast and complex chemical space by considering different molecular modifications iteratively. The MCTS simulates the potential outcomes of these modifications, allowing for efficient decision making.

Altogether, this architecture integrates the transformer’s generative capabilities with the MCTS’s exploitation and exploration techniques in order to optimize the discovery of promising drug candidates that can meet multiple optimization goals. This architecture is designed to make sure the generated molecules are valid, show high bioactivity against target receptors, and can be administered orally.

### 4.3. Encoder–Decoder Transformer

At the core of our drugAI engine is the transformer model, as shown in Figure 3. We selected this deep-learning model for the following reasons:

- Transformers excel at modeling sequential data. We see molecule generation as a machine translation task that needs to follow a sequence-to-sequence model (seq-to-seq).
- Transformers are parallelizable, and this makes it efficient to parallelize the training and inference steps against Graphics Processing Units (GPUs) and Tensor Processing Units (TPUs).
- Transformers can capture distant or long-range contexts and dependencies in the data between distant positions in the input or output sequences. Thus, longer connections can be learned, which makes it ideal for learning and capturing amino acid sequences whose residues can be hundreds or even thousands in length.
- Transformers make no assumptions about the temporal/spatial relationships across the data.

For machine translation tasks that need to be modeled as seq-to-seq, the suitable transformer architecture is the encoder–decoder model. The encoder consists of encoding layers that process the amino acids iteratively one layer after another, while the decoder consists of decoding layers that iteratively process the encoder’s output as well as the decoder output’s SMILES strings in an auto-regressive manner.

The purpose of having an encoder layer is to generate a context representation of the protein (protein context), where each amino acid residue is represented by a “protein vector”. It combines information from other amino acid residues via the self-attention mechanism. On the other hand, the decoder is responsible for generating the corresponding atoms that make up the small molecule in the SMILES notation. Each of the decoder layers consists of two sub-layers: (1) cross attention to incorporate the outputs of the encoder (also known as protein context); and (2) self-attention, which implements a teacher-forcing mechanism, feeding the decoder model with the previously predicted atoms to predict the probability distributions of the next atom from the vocabulary of SMILES notations. Both the cross-attention and self-attention layers also include an additional feed-forward layer and layer normalization for further processing of the outputs.

The building blocks of the transformer are self-attention, where each attention unit learns three weight matrices: the query weights $W_{Q}$, the key weights $W_{K}$, and the value weights $W_{V}$.

For each residue *i*, the input protein representation $x_{i}$ is multiplied with each of the three weight matrices to produce a query vector $q_{i\;}=x_{i}W_{Q}$, a key vector $k_{i\;}=x_{i}W_{K}$, and a value vector $v_{i\;}=x_{i}W_{V}$.

Attention weights are calculated using the query and key vectors: the attention weight $a_{ij\;}$ from amino acid *i* to amino acid *j* is the dot product between $q_{i}$ and $k_{j}$. The attention weights are divided by $\sqrt{d_{k}}$ to stabilize gradients during training, and pass through a softmax layer, which normalizes the weights.

In summary, self-attention can be represented by the formula

$$Attention\;\left(Q,K,V\right)=softmax\frac{{QK}^{T}}{\sqrt{d_{k}}}$$

A set of ($W_{Q}$, $W_{K}$, $W_{V}$) matrices is called an attention head and each layer in a transformer model can have multiple attention heads. While each attention head attends to the amino acids that are relevant to each residue, multiple attention heads allow the model to do this for different definitions of relevance.

### 4.4. SMILES Decoding Strategies

#### 4.4.1. Greedy Search

The greedy algorithm is one of the most common decoding algorithms, especially in Natural Language Processing. For drug design purposes, it simply generates one SMILE token at a time, iteratively. At each step, the model predicts the next token in the sequence based on the context of previously generated tokens. Note that in greedy decoding, the model selects the token with the highest probability as the next token to generate. This means that at each step, the model does not consider the global context or explore alternative token choices but simply chooses the most likely token according to its learned probabilities. This process continues until a predefined end-of-sequence token (e.g., <eos> for “end of SMILES”) is generated or a maximum sequence length is reached. While greedy decoding is straightforward and computationally efficient, it may not always produce the best possible sequence, as it tends to favor local optimal choices at each step.

#### 4.4.2. Beam Search

Beam search is another commonly used decoding algorithm. This decoder generates a set of candidate SMILES tokens for the next step in the sequence based on the current state (also known as teacher forcing). Each candidate is assigned a score based on its likelihood that is typically calculated using a combination of the model’s output probabilities and a length normalization factor. The candidates with the highest scores are retained, while the rest are discarded. The retained candidates become the new set of states for the next step. These states are expanded further by generating new candidates for the subsequent step until a predefined end-of-sequence token is generated or a maximum sequence length is reached.

#### 4.4.3. Monte Carlo Tree Search

The Monte Carlo Tree Search (MCTS) is a heuristic search algorithm that is often used in decision-making processes, particularly in the domain of artificial intelligence and game-playing software. It is commonly employed in software designed to play board games and other strategy games that require complex and branching decision trees to explore all possible moves and outcomes exhaustively. As the name suggests, it uses random sampling for deterministic problems that are difficult to solve using other traditional approaches due to the vast search space. The popularity of the algorithm increased after Google’s DeepMind adopted it to build a program called AlphaGo [34] that became the first computer Go program to beat a professional human Go player.

**Figure 4:**
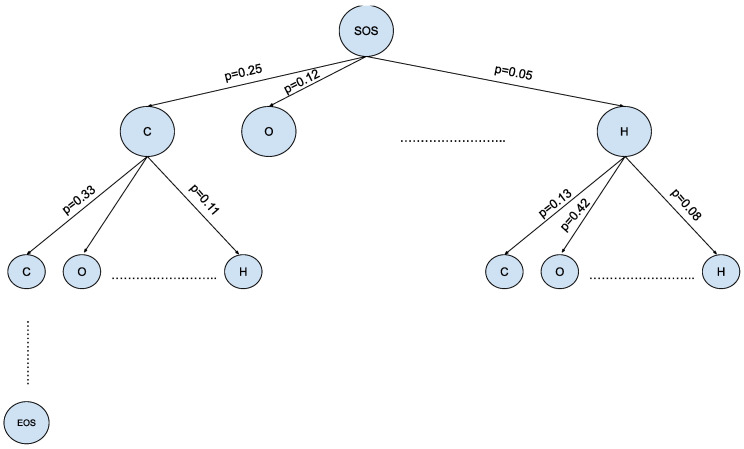
Vast chemical space in the tree structure format—$V^{d}$ where V is the size of the SMILES vocabulary and d is the max depth of small molecules in SMILES notation.

The MCTS offers a promising solution for navigating the expansive and intricate landscape of chemical space in the context of molecule generation, as shown in Figure 4. This algorithmic framework, originally developed for game-playing AI, has found a novel application in the field of Natural Language Processing [19]. The MCTS leverages the principles of exploration and exploitation to efficiently sample and evaluate molecular candidates. By iteratively building a tree of possible chemical sequences and selecting the most promising branches evaluated via a reward function, the MCTS enables the exploration of diverse regions within the vast chemical space while maintaining a focus on regions likely to yield valuable molecules. This innovative approach holds the potential to revolutionize drug discovery, materials science, and other domains by aiding researchers in the rapid and intelligent exploration of uncharted territories within the complex realm of molecular design.

**Figure 5:**
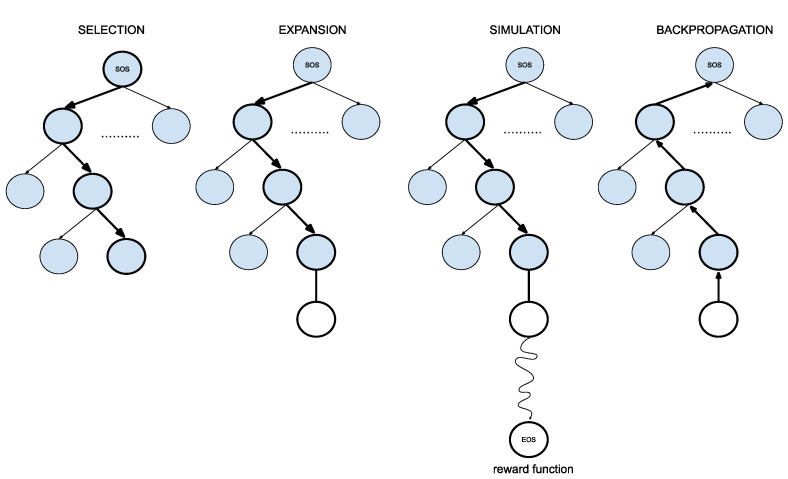
Four steps of Monte Carlo Tree Search (adapted from [20]).

To apply this powerful approach in drug design, we modified the original MCTS algorithm as described below and provide a summary in Figure 5:

1. Selection
  The MCTS traverses the SMILES tree structure from the root node using a strategy called the Upper Confidence Bound (UCB) to optimally select the subsequent nodes with the highest estimated value of UCB. Values derived by UCB balance the exploration-exploitation trade-off, and during the tree traversal, a node is selected based on some parameters that return the maximum value. The formula of UCB is described as follows:
  $$UCB=\frac{r_{i}}{n_{i}}+c\sqrt{\frac{lnN_{i}}{n_{i}}}$$
  $r_{i}\;$is total cumulative rewards.
  $n_{i}\;$is the number of simulations for the node considered after i-th move.
  $N_{i}$ is the total number of simulations after i-th move.
  c is the exploration parameter (default value set to 2).
  In summary, the first term of the equation will help determine when the MCTS should prioritize making the most of what it knows (exploitation) and the second term of the equation will determine when it should focus on trying out new options (exploration). This ensures that the algorithm is balanced between ensuring that it explores new possibilities and also exploiting known good choices.
2. Expansion
  During the traversal of a SMILES tree as part of the selection process in the Monte Carlo Tree Search (MCTS), the child node that yields the highest value from the equation will be chosen for further exploration. If this selected node is also a leaf node and not a terminal node, the MCTS will proceed with the expansion process. This involves creating all possible children of this leaf node based on the SMILES vocabulary.
3. Simulation
  The posterior distribution is derived by using the distribution supplied by the decoder as an informative prior (in contrast to using uniform distribution as a prior where a large proportion of the posterior samples are in the invalid form).
4. Back propagation
  When the terminal node is reached, the complete SMILES string can be finalized by concatenating all the traversed and simulated nodes from the root until the terminal and the reward is calculated by running the reward function based on the newly constructed SMILES string. Thus, the MCTS needs to update the traversed nodes with this new reward by performing a back-propagation process where it back-propagates from the selected leaf node as a result of step 2 all the way back up to the root node. During this process, the number of simulations stored in each node is also incremented.

In our emphasis on developing an effective reward function tailored for the drug design use case, we considered various options that could be employed as the reward function. Below, we describe the choices we used for the reward function.

1. Valid SMILES
  This binary variable is a straightforward check that assesses the validity of the newly generated SMILES string resulting from the simulation. The check is carried out by executing a basic function provided by RDKit, a toolkit commonly used in cheminformatics and drug discovery [35].
2. Lipinski’s Rule of Five (Ro5)
  Another binary variable that checks to see if the newly constructed SMILES string passes all the 5 conditions set forth in the Ro5 [22].
3. Quantitive Estimation of Drug-likeness (QED)
  A floating-point variable that reflects the underlying distribution of molecular properties. This metric is intuitive, transparent, and straightforward to implement in many practical settings and allows compounds to be ranked by their relative merit. Medicinal chemists often consider a compound to exhibit characteristics and properties typically desired in drug candidates if the correlation coefficient of the QED value falls within the range of 0.5 to 0.6 [23].
4. Binding Affinity (kcal/mol)
  A floating-point variable that refers to the strength by which two molecules interact or bind. The smaller its value, the greater the affinity between two molecules. This binding affinity score is generated by AutoDock Vina [36], a commonly used open-source program for doing molecular docking.

## 5. Conclusions and Future Perspectives

In conclusion, drugAI outperformed models using greedy and beam searches regarding the validity, QED, and adherence of the generated molecules to RO5. We further improved the drug design capabilities by adding a binding affinity reward function in drugAI’s reinforcement learning. This proof of concept, combining the Encoder–Decoder Transformer architecture with the flexibility of the MCTS-based reinforcement learning, has the potential to significantly improve the quality of generated drugs by incorporating even more reward functions.

Future research could enhance the reinforcement learning of drugAI by supplementing the model with additional functions, such as the following:

1. pChEMBL values, including pKi, pKd, pIC50, or pEC50 [37];
2. ADMET-related properties, such as acute oral toxicity, Ames mutagenicity, and Caco-2 permeability;
3. Adherence to Oprea’s rules of drug-likeness [38];
4. Avoidance of functional groups with toxic, reactive, or otherwise undesirable moieties defined by the REOS (Rapid Elimination of Swill) rules [39].

Such a multi-objective optimization reinforcement learning approach could potentially yield valuable molecules. This approach would enable the model to simultaneously optimize multiple properties or functions rather than focusing on a single objective. By optimizing across multiple criteria, it may be possible to create molecules that are not only effective as potential drugs but also meet various safety and efficacy requirements. However, the success of this approach will depend on the specific objectives and constraints defined, as well as the quality of data used to train the model.

## Figures and Tables

**Table 1:** Summary of the GuacaMol evaluations of the SMILES strings generated by three different decoding algorithms. The reported values represent the averages of ten independent runs. Significant differences are denoted by different letters, as determined by the Kruskal–Wallis test (*p* < 0.05). The values based on a 95% confidence interval are presented in parentheses.

Benchmark	DrugAI	Greedy	Beam (K = 2)
Validity	1.00 ^a^ (1)	0.83 ^b^ (0.82–0.84)	0.62 ^c^ (0.61–0.63)
Uniqueness	0.84 ^a^ (0.83–0.85)	0.87 ^a^ (0.86–0.88)	0.37 ^b^ (0.36–0.38)
Novelty	1.00 ^a^ (1)	1.00 ^a^ (1)	0.47 ^b^ (0.46–0.48)
Mean QED	0.73 ^a^ (72.95–73.05)	0.41 ^b^ (0.41)	0.18 ^c^ (0.18)

**Table 2:** Molecules generated by drugAI against SARS-CoV-2 M^pro^ target protein.

Generated Molecule (SMILES)	Validity	Adherence to RO5	Binding Affinity (kcal/mol)	QED Score
O=C1c2cc(N3CCNCC3)ccc2C(OC2CCc3ccccc32)c2ccccc21	1	1	−9.87	0.69
O=C1c2cc(N3CCN(c4cnc5ncccc5c4)CC3)ccc2CCCc2ccccc21	1	1	−9.59	0.46
O=C1c2cc(N3CCNCC3)cc(-c3cccc(/C=C/C(=O)Nc4ccccc4)c3)c2C(=O)N1	1	1	−9.56	0.41
O=C1c2cc(N3CCNC(c4ncc(C(F)(F)F)cc4Cl)CC3)ccc2COC1O	1	1	−9.45	0.74
O=C1c2cc(N3CCNCC3)ccc2OC(COc2cccc([N+](=O)[O-])c2)Cc2ccccc21	1	1	−9.39	0.46
O=C1c2cc(N3CCNCC3)nc-c3ccc(C(=O)c4cc(F)cc(F)c4)cc3c2CCC1=O	1	1	−9.39	0.56
O=C1c2cc(N3CCNC4C3CC5CC(C4)OC5)ccc2C(=O)c2ccc(Cl)cc2N1	1	1	−9.30	0.61
O=C1c2cc(N3CCNCC3)nc(-c3cccc(C(F)(F)F)c3)c2CCc2ccccc21	1	1	−9.24	0.64
O=C1c2cc(N3CCNCC3)ccc2C(OC2Cc3ccccc3C2)=C2C(=O)CCC(O)C(F)(F)C21	1	1	−9.22	0.68
O=C1c2cc(N3CCNCC3)ccc2OC/C1=C(\O)c1cccc(-c2ccncc2)c1	1	1	−9.21	0.50
Average	1	1	−9.42	0.58

## Data Availability

The complete code used in this research is publicly available at https://github.com/dangjaya/drugAI. A standalone more user-friendly executable version of drugAI is available for download at https://atamianlab.com/software/.

## Supplementary Materials

The following supporting information can be downloaded at: https://www.mdpi.com/article/10.3390/ph17020161/s1, Table S1: Annotation of the molecules generated by drugAI.

## References

[1] Walters W.P. (2018). Virtual chemical libraries: Miniperspective. J. Med. Chem..

[2] Dreiman G.H., Bictash M., Fish P.V., Griffin L., Svensson F. (2021). Changing the HTS paradigm: AI-driven iterative screening for hit finding. SLAS Discov..

[3] Senger M.R., Fraga C.A., Dantas R.F., Silva F.P. (2016). Filtering promiscuous compounds in early drug discovery: Is it a good idea?. Drug Discov. Today.

[4] Gupta M.N., Alam A., Hasnain S.E. (2020). Protein promiscuity in drug discovery, drug-repurposing and antibiotic resistance. Biochimie.

[5] Frantz S. (2005). Drug discovery: Playing dirty. Nature.

[6] Lin X., Li X., Lin X. (2020). A review on applications of computational methods in drug screening and design. Molecules.

[7] Sharma V., Wakode S., Kumar H., Sharma N., Ojha H., Raghav P.K., Goyal R.K. (2021). Structure- and ligand-based drug design: Concepts, approaches, and challenges. Chemoinformatics and Bioinformatics in the Pharmaceutical Sciences.

[8] Salo-Ahen O.M., Alanko I., Bhadane R., Bonvin A.M., Honorato R.V., Hossain S., Juffer A.H., Vanmeert M. (2020). Molecular dynamics simulations in drug discovery and pharmaceutical development. Processes.

[9] Cheng Y., Gong Y., Liu Y., Song B., Zou Q. (2021). Molecular design in drug discovery: A comprehensive review of deep generative models. Brief. Bioinform..

[10] Xie W., Wang F., Li Y., Lai L., Pei J. (2022). Advances and challenges in de novo drug design using three-dimensional deep generative models. J. Chem. Inf. Model..

[11] Brown T., Mann B., Ryder N., Subbiah M., Kaplan J.D., Dhariwal P., Neelakantan A., Amodei D. Language models are few-shot learners. Proceedings of the Advances in Neural Information Processing Systems 33 (NeurIPS 2020).

[12] Devlin J., Chang M.W., Lee K., Toutanova K. (2018). Bert: Pre-training of deep bidirectional transformers for language understanding. arXiv.

[13] Chowdhary K.R. (2020). Natural language processing. Fundamentals of Artificial Intelligence.

[14] Kell D.B., Samanta S., Swainston N. (2020). Deep learning and generative methods in cheminformatics and chemical biology: Navigating small molecule space intelligently. Biochem. J..

[15] Sutskever I., Vinyals O., Le Q.V. Sequence to sequence learning with neural networks. Proceedings of the Advances in Neural Information Processing Systems 27 (NIPS 2014).

[16] Vaswani A., Shazeer N., Parmar N., Uszkoreit J., Jones L., Gomez A.N., Kaiser L., Polosukhin I. Attention is all you need. Proceedings of the Neural Information Processing Systems 30 (NIPS 2017).

[17] Chickering D.M. (2002). Optimal structure identification with greedy search. J. Mach. Learn. Res..

[18] Wiseman S., Rush A.M. (2016). Sequence-to-sequence learning as beam-search optimization. arXiv.

[19] Leblond R., Alayrac J.B., Sifre L., Pislar M., Lespiau J.B., Antonoglou I., Simonyan K., Vinyals O. (2021). Machine translation decoding beyond beam search. arXiv.

[20] Chaffin A., Claveau V., Kijak E. (2021). PPL-MCTS: Constrained textual generation through discriminator-guided MCTS decoding. arXiv.

[21] Gilson M.K., Liu T., Baitaluk M., Nicola G., Hwang L., Chong J. (2016). BindingDB in 2015: A public database for medicinal chemistry, computational chemistry and systems pharmacology. Nucleic Acids Res..

[22] Lipinski C.A. (2004). Lead-and drug-like compounds: The rule-of-five revolution. Drug Discov. Today Technol..

[23] Bickerton G.R., Paolini G.V., Besnard J., Muresan S., Hopkins A.L. (2012). Quantifying the chemical beauty of drugs. Nat. Chem..

[24] Brown N., Fiscato M., Segler M.H., Vaucher A.C. (2019). GuacaMol: Benchmarking models for de novo molecular design. J. Chem. Inf. Model..

[25] Benet L.Z., Hosey C.M., Ursu O., Oprea T.I. (2016). BDDCS, the Rule of 5 and drugability. Adv. Drug Deliv. Rev..

[26] Ang D., Kendall R., Atamian H.S. (2023). Virtual and In Vitro Screening of Natural Products Identifies Indole and Benzene Derivatives as Inhibitors of SARS-CoV-2 Main Protease (Mpro). Biology.

[27] Harshvardhan G.M., Gourisaria M.K., Pandey M., Rautaray S.S. (2020). A comprehensive survey and analysis of generative models in machine learning. Comput. Sci. Rev..

[28] Martinelli D.D. (2022). Generative machine learning for de novo drug discovery: A systematic review. Comput. Biol. Med..

[29] Grechishnikova D. (2021). Transformer neural network for protein-specific de novo drug generation as a machine translation problem. Sci. Rep..

[30] Latif S., Cuayáhuitl H., Pervez F., Shamshad F., Ali H.S., Cambria E. (2023). A survey on deep reinforcement learning for audio-based applications. Artif. Intell. Rev..

[31] Mouchlis V.D., Afantitis A., Serra A., Fratello M., Papadiamantis A.G., Aidinis V., Lynch I., Greco D., Melagraki G. (2021). Advances in de novo drug design: From conventional to machine learning methods. Int. J. Mol. Sci..

[32] Fan J., Fu A., Zhang L. (2019). Progress in molecular docking. Quant. Biol..

[33] Parenti M.D., Rastelli G. (2012). Advances and applications of binding affinity prediction methods in drug discovery. Biotechnol. Adv..

[34] Silver D., Huang A., Maddison C.J., Guez A., Sifre L., van den Driessche G., Schrittwieser J., Antonoglou I., Panneershelvam V., Lanctot M. (2016). Mastering the game of Go with deep neural networks and tree search. Nature.

[35] RDKit: Open-Source Cheminformatics. https://www.rdkit.org.

[36] Trott O., Olson A.J. (2010). AutoDock Vina: Improving the speed and accuracy of docking with a new scoring function, efficient optimization, and multithreading. J. Comput. Chem..

[37] Bento A.P., Gaulton A., Hersey A., Bellis L.J., Chambers J., Davies M., Kruger F.A., Overington J.P. (2014). The ChEMBL bioactivity database: An update. Nucleic Acids Res..

[38] Oprea T.I. (2020). Property distribution of drug-related chemical databases. J. Comput. Aided Mol. Des..

[39] Walters W.P., Namchuk M. (2003). Designing screens: How to make your hits a hit. Nat. Rev. Drug Discov..

